# A comprehensive approach to adolescent suicide prevention: insights from a narrative review perspective

**DOI:** 10.3389/fpsyg.2025.1612067

**Published:** 2025-11-07

**Authors:** Valentina Baldini, Martina Gnazzo, Giorgia Varallo, Matteo Di Vincenzo, Maristella Scorza, Christian Franceschini, Diana De Ronchi, Andrea Fiorillo, Giuseppe Plazzi

**Affiliations:** 1 Department of Biomedical and Neuromotor Sciences, University of Bologna, Bologna, Italy; 2 Department of Biomedical, Metabolic and Neural Sciences, University of Modena and Reggio Emilia, Modena, Italy; 3 Department of Psychiatry, University of Campania "Luigi Vanvitelli", Naples, Italy; 4 Department of Medicine and Surgery, University of Parma, Parma, Italy; 5 IRCCS Istituto Delle Scienze Neurologiche di Bologna, Bologna, Italy

**Keywords:** suicide, adolescents, suicidal behavior, intervention strategies, NSSI behavior

## Abstract

Adolescent suicide remains a critical global public health challenge, with rising incidence rates necessitating urgent action. This narrative review presents a comprehensive strategy for reducing suicide risk among adolescents by synthesizing current evidence on risk factors, early detection, intervention, and prevention. Key risk factors include mental health disorders, substance use, trauma, and social determinants such as bullying and family dynamics. Schools, healthcare systems, and community-based initiatives play a vital role in early detection and intervention. This review examines evidence-based strategies, including universal screening, expanded access to mental health care, and targeted interventions such as cognitive behavioral therapy (CBT) and dialectical behavioral therapy (DBT). It also explores the impact of public health campaigns, policy reforms, and technology-driven approaches on strengthening adolescent mental health awareness and resilience. A multi-sectoral, culturally sensitive approach—one that prioritizes the voices of adolescents, engages families and promotes health equity—is essential for reducing the risk of suicide and enhancing youth well-being globally. This review underscores the urgent need for enhanced suicide prevention policies, improved mental health service accessibility, and the integration of digital tools in adolescent care. Addressing research gaps through longitudinal studies and real-world implementation trials will be key in shaping future strategies to reduce adolescent suicide rates globally.

## 1 Introduction

Adolescence is a crucial stage of development characterized by significant physical, emotional, and social changes. During this period, individuals navigate vital milestones in identity formation, peer relationships, and academic progress (World Health Organization, 2021). While these experiences foster growth and resilience, they may also contribute to vulnerabilities that can lead to mental health issues. Among the latter, suicide stands out as a leading cause of death among adolescents, ranking as the third leading cause of death for individuals aged 10 to 19 worldwide (Bridge et al., 2022). In this review, adolescence was defined as the age range 10–19 years, following the World Health Organization’s medical definition (World Health Organization, 2021). We did not include individuals aged 18–21 years, often called ‘late adolescents’ or ‘emerging adults,’ to stay consistent with this definition and ensure comparability across studies. The need to address adolescent suicide is underscored by its devastating impact on families, communities, and society.

The etiology of adolescent suicide is multifactorial, involving a complex interplay of biological, psychological, and environmental factors. Neurobiological research has linked suicidal behavior to dysregulation of serotonergic systems, increased activity of the hypothalamic–pituitary–adrenal axis, and impaired executive functioning (Romer, 2010). Moreover, adolescents show heightened vulnerability to stressors such as interpersonal conflicts, academic pressures, and social media influences, which can exacerbate feelings of isolation and affect their mood and sleep (Baldini et al., 2023; Baldini et al., 2024; Alqueza et al., 2023; Baldini et al., 2025). Furthermore, the rising prevalence of cyberbullying and negative portrayals of mental health on online platforms have emerged as modern contributors to the risk of adolescent suicide (Hamm et al., 2015).

Despite decades of research and advocacy, significant barriers persist in preventing adolescent suicide. The stigma surrounding mental health continues to obstruct help-seeking behaviors, with many adolescents hesitating to disclose emotional distress or seek mental health services (Miche et al., 2020). Disparities in healthcare access, particularly among marginalized and underserved populations, further complicate the issue (Clayborne et al., 2019). These barriers underscore the necessity for systemic change and innovative prevention strategies.

Addressing adolescent suicide requires a comprehensive approach that considers developmental, cultural, and contextual factors specific to this age group. School-based mental health programs have proven effective in raising awareness and detecting suicide risk early; family-centered interventions provide critical support in fostering communication and emotional resilience (Mellor, 2014; Schachter et al., 2008). Additionally, community-wide initiatives, including responsible media guidelines for reporting on suicide and campaigns designed to reduce stigma, are crucial for creating supportive environments (Centers for Disease Control and Prevention, National Center for Injury Prevention and Control, 2021).

The emergence of digital health tools, including smartphone applications and telehealth platforms, creates new opportunities for suicide prevention. These technologies offer discreet and accessible support to adolescents, especially in rural or resource-limited areas (Luxton et al., 2012). However, concerns regarding data privacy, digital literacy, and equitable access must be addressed to ensure their effectiveness and reach (Torok et al., 2019).

This narrative review provides a comprehensive synthesis of current evidence on adolescent suicide risk, focusing on four key areas: risk factors, early detection, intervention, and prevention. By examining the most relevant findings in each domain, this review aims to guide clinicians, educators, policymakers, and researchers in developing targeted strategies and fostering supportive environments that enhance adolescent mental health. The review also highlights recent research advancements, identifies persistent knowledge gaps, and outlines best practices to inform future directions in suicide prevention among adolescents.

## 2 Methods

This narrative review synthesizes existing evidence on the risk factors and prevention strategies for adolescent suicide. The methodology follows established guidelines for conducting narrative reviews, focusing on a comprehensive exploration and critical analysis of the literature without imposing systematic limitations. We opted for a narrative review to provide an integrated and critical perspective on existing literature, including diverse study designs and theoretical perspectives that would not be captured in a systematic review. This approach allows for a broader synthesis of findings and identification of knowledge gaps beyond the constraints of systematic methodologies.

### 2.1 Search strategy

A comprehensive search strategy was employed to identify relevant articles from peer-reviewed journals, books, and authoritative reports. This search utilized electronic databases, including PubMed, PsycINFO, Scopus, and Web of Science. The search terms comprised various combinations of the following keywords: “adolescent suicide,” “suicide risk,” “prevention strategies,” “mental health,” “cyberbullying,” “family interventions,” and “school-based programs.” Boolean operators (AND/OR) were applied to refine the search results. The literature search was conducted between January and July 2024, with a final update in March 2025 prior to submission. All databases were searched from their inception up to March 2025. To ensure a thorough approach, the search was enhanced by manually reviewing the reference lists of included articles. Grey literature, such as reports from the World Health Organization (WHO) and the Centers for Disease Control and Prevention (CDC), was also taken into consideration.

### 2.2 Inclusion and exclusion criteria

Studies were included in the review if they fulfilled the following criteria:

1. Addressed adolescent populations (ages 10–19);
2. Emphasized suicide risk factors, prevention strategies, or interventions;
3. Were published in English;
4. Were peer-reviewed or acknowledged as authoritative grey literature.

Studies were not included if they:

1. Concentrated solely on adult or pediatric populations, excluding the adolescent age range;
2. Did not provide sufficient detail on suicide risk or prevention;
3. Were opinion pieces, editorials, or commentaries which did not support empirical evidence.

### 2.3 Data extraction and synthesis

The authors independently collected data, focusing on study characteristics and key findings related to suicide risk factors and prevention strategies. To maintain the narrative format, the extracted data were synthesized qualitatively. Emphasis was placed on identifying patterns, themes, and gaps in the literature instead of conducting a quantitative meta-analysis.

### 2.4 Quality assessment

While formal quality appraisal is not invariably necessary for narrative reviews, an informal assessment was undertaken to prioritize high-quality studies. The factors taken into account included methodological rigor and relevance to the review topic. Studies published in high-impact journals or endorsed by reputable organizations were afforded greater significance in the synthesis.

## 3 Results

**Figure 1:**
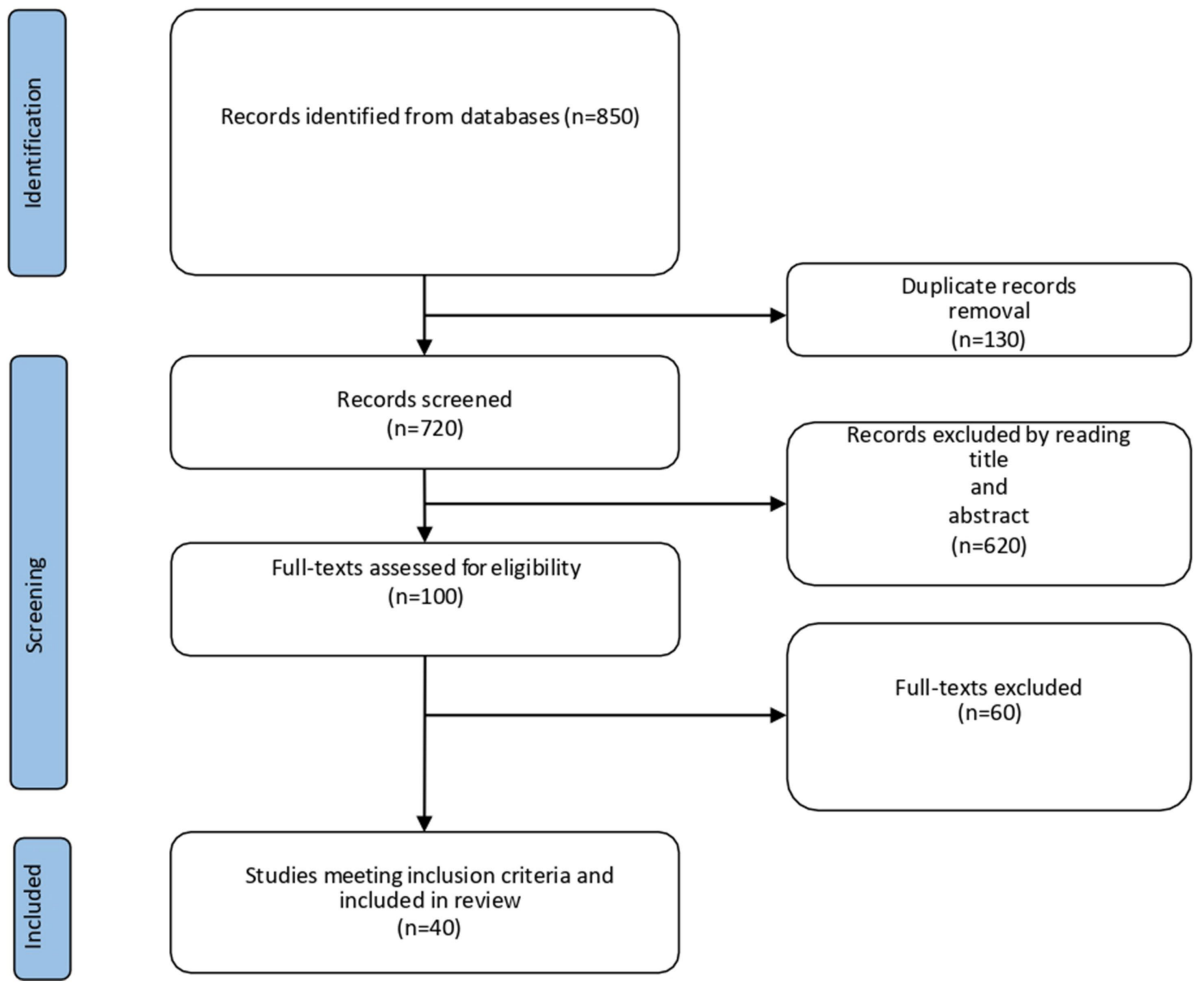
Flowchart describing the study selection process.

**Figure 2:**
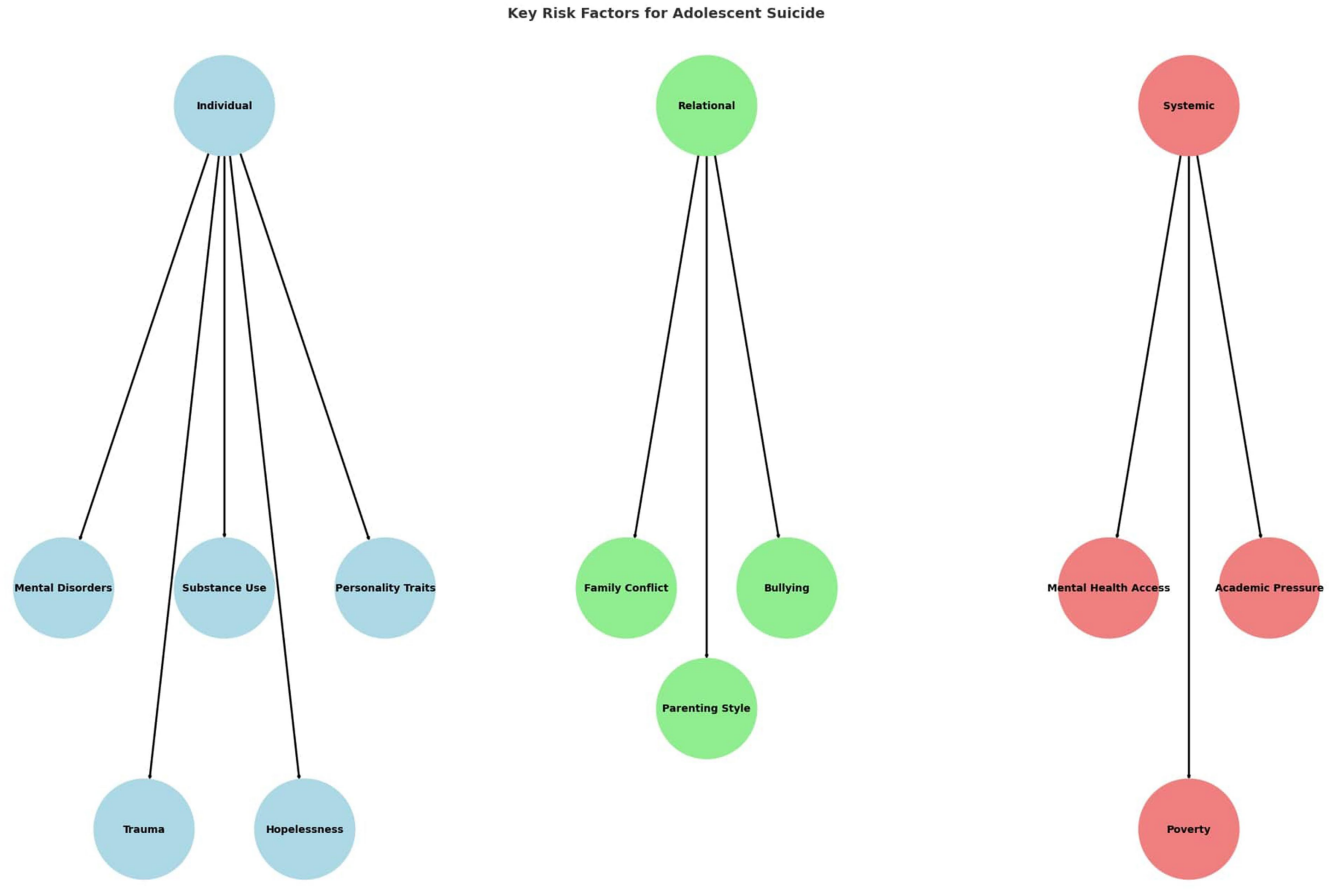
Key risk factors for adolescent suicide grouped into three domains: individual, relational, and systemic. Individual factors include mental disorders, substance use, personality traits, trauma, and hopelessness. Relational factors comprise family conflict, bullying, and parenting style. Systemic factors involve limited mental health access, academic pressure, and poverty.

The literature search initially identified approximately 850 records. After removing duplicates and screening titles/abstracts, 720 papers were assessed for eligibility. Following full-text review, 70 studies were included in this narrative synthesis: 40 observational studies, 10 intervention trials, 15 reviews, and 5 institutional reports. The study selection process is presented in Figure 1. A selection of representative studies is presented in Table 1, while the full set of 40 included articles is discussed throughout the narrative synthesis. Key risk factors identified across the included studies are summarized in Figure 2, organized into individual, relational, and systemic domains.

**Table 1:** Summary of selected representative studies included in this narrative synthesis.

Author (Year)	Country	Study type	Sample (*N*, age)	Focus	Main findings
Franklin et al., 2017	USA	Meta-analysis	365 studies, adolescents and adults	Suicide risk factors	Depression, prior attempts, and impulsivity identified as strongest predictors
Ho et al., 2022	China	Observational	*N* = 1,245 adolescents (12–18)	Pubertal timing	Early puberty associated with higher suicide risk
Baldini et al., 2024	Italy	Review/meta-analysis	Adolescents 10–19	Sleep disturbances	Insomnia and nightmares consistently linked with suicidal ideation
Reupert et al., 2013	Australia	Review	Children/adolescents	Parental mental illness	Offspring experience caregiving burden, increasing psychological vulnerability
Hong et al., 2015	USA	Observational	*N* = 5,000 adolescents	Bullying	Victims at higher risk of suicidal ideation and attempts
Zhou et al., 2023	China	Observational	*N* = 3,200 adolescents	Cyberbullying	Cybervictimization strongly associated with depression and suicidal ideation
Doty et al., 2022	Multi-country	Observational	*N* = 12,000 adolescents	Socioeconomic status	Lower SES linked with higher suicide attempts
Miller-Graff et al., 2016	USA	Observational	*N* = 900 adolescents	Family conflict	High exposure predicted suicidality beyond depression
Liu et al., 2024	China	Observational	*N* = 2,700 adolescents	SES and NSSI	Low SES and family adversity associated with self-harm and suicide risk
Mehlum et al., 2014	Norway	RCT	*N* = 77 adolescents (12–18)	DBT intervention	DBT reduced self-harm and suicidal ideation
McCauley et al., 2018	USA	RCT	*N* = 173 adolescents (12–18)	DBT vs. supportive therapy	DBT significantly reduced suicide attempts
Torok et al., 2019	Australia	Review	—	Gatekeeper training	Training improved recognition of suicide risk in adolescents
Mirkovic et al., 2020	France	Observational	*N* = 300 adolescents inpatients	Predictors of suicide attempts	Previous attempts strongly predicted recurrence at follow-up
Carballo et al., 2020	Spain	Review	Children/adolescents	Psychosocial risk factors	Family conflict, bullying, and psychiatric comorbidities identified as major risks
Klonsky et al., 2016	USA	Review/meta-analysis	Adolescents and adults	Suicidal ideation vs. attempts	Identified distinct predictors differentiating ideation from attempts
Mortier et al., 2018	Belgium	Meta-analysis	College students and adolescents	Prevalence	High prevalence of suicidal thoughts and behaviors across countries
Keles and Idsoe, 2018	Norway	Meta-analysis	Adolescents in schools	CBT-based prevention	Group CBT effective for reducing depression and suicidal ideation
Santamarina-Perez et al., 2020	Spain	RCT	*N* = 60 adolescents (12–18)	DBT in community clinic	Adapted DBT effective in reducing suicidal behaviors
Emslie et al., 2010	USA	RCT (TORDIA)	*N* = 334 adolescents with resistant depression	Treatment-resistant depression	Combination of medication and CBT more effective than medication switch alone
Müller et al., 2024	Germany	RCT	*N* = 120 children/adolescents	Family-based preventive intervention	Improved coping and reduced suicidal ideation in offspring of parents with severe mental illness

The table is not exhaustive; rather, it highlights a range of observational, interventional, and review/meta-analytic studies discussed in the manuscript to illustrate the diversity of designs and findings among the 40 studies included.

### 3.1 Risk factors for adolescent suicide

Adolescent suicide results from a complex interaction of vulnerabilities across various domains. Risk factors can be grouped into individual, relational, and socio-ecological categories, each having a distinct yet interconnected impact on suicidal thoughts and actions. Personal vulnerabilities like psychiatric conditions and impulsivity often combine with family relationships, peer influences, and wider societal factors such as socioeconomic status or the school environment. The following sections offer an overview of these areas, emphasizing the multifaceted nature of adolescent suicide risk.

#### 3.1.1 Individual factors

Mental health disorders serve as the most substantial individual-level contributors to adolescent suicide. In particular, major depressive disorder (MDD) is consistently recognized as a principal risk factor. Adolescents with a diagnosis of MDD show a markedly increased vulnerability to suicidal ideation and behavior, with two studies suggesting a two- to three-fold higher risk compared to those without depression (Clayborne et al., 2019; Doty et al., 2022).

Additionally, anxiety disorders, notably generalized anxiety disorder, and social anxiety disorder demonstrate a strong correlation with suicide risk. Externalizing disorders, such as conduct disorder and attention-deficit/hyperactivity disorder, also display significant associations with increased suicide risk, particularly among male adolescents (Mirkovic et al., 2020).

Substance use disorders significantly elevate the risk by impairing judgment, reducing inhibitions, and amplifying impulsivity. The use of alcohol and drugs is particularly alarming due to its frequent co-occurrence with other mental health conditions, which exacerbate feelings of hopelessness or despair (Substance Abuse and Mental Health Services Administration, 2022).

Certain personality traits, such as perfectionism and heightened levels of neuroticism, are associated with an increased susceptibility to suicidal behavior. Adolescents who exhibit impulsivity and diminished emotional regulation are more likely to act on suicidal thoughts, particularly during times of significant distress (Gallagher et al., 2014). Impulsivity serves a pivotal function in the transition from suicidal ideation to action, underscoring the necessity for interventions that enhance self-control and coping strategies.

Exposure to trauma and adverse childhood experiences (ACEs), such as physical, emotional, or sexual abuse, significantly increases the risk of adolescent suicide. ACEs disrupt normal developmental pathways, resulting in chronic stress, unhelpful coping strategies, and changes in neurobiological systems, including the HPA axis (Hong et al., 2015). Adolescents with a history of ACEs frequently experience ongoing feelings of worthlessness, mistrust, and challenges in forming healthy relationships, all of which heighten the risk of suicide.

Although ACEs typically originate from relational settings such as family conflict, neglect, or abuse, we categorized them as individual factors because their long-term effects mainly show through psychological and neurobiological changes (e.g., stress-response dysregulation, impaired emotional regulation). In this way, ACEs illustrate how relational hardships turn into individual vulnerabilities that increase suicide risk.

Hopelessness, a cognitive state characterized by negative expectations about the future, is a well-documented predictor of suicide among adolescents. Cognitive distortions, such as black-and-white thinking and overgeneralization, intensify feelings of inadequacy and despair (Carballo et al., 2020). Adolescents who struggle with problem-solving and adaptive coping skills find it more challenging to manage life stressors, which further increases their vulnerability (Kang et al., 2012).

Self-harm behaviors, such as cutting or burning, are both risk factors and warning signs for suicide. Adolescents who engage in self-harm often use it as a maladaptive coping mechanism to manage overwhelming emotions, and it also serves as a predictor of future suicide attempts (Kim, 2021). Similarly, a history of previous suicide attempts is one of the strongest predictors of subsequent suicide, highlighting the importance of early intervention and ongoing monitoring (Klonsky et al., 2016).

In summary, the factors contributing to adolescent suicide risk are multifaceted and involve an interplay of biological, psychological, and developmental elements. While these factors are deeply personal, relational and systemic influences often amplify their effects, underscoring the need for comprehensive, individualized prevention strategies.

#### 3.1.2 Relational factors

Family relationships are crucial for adolescent development, and disruptions in family dynamics are strongly linked to an increased risk of suicide. Adolescents who face high levels of family conflict, parental neglect, or inconsistent caregiving are more likely to develop mental health issues and engage in suicidal behaviors (Mortier et al., 2018). Authoritarian parenting styles, characterized by strict rules and a lack of emotional warmth, often result in feelings of inadequacy and rejection among adolescents (Duan et al., 2022).

Parental mental health affects adolescent suicide risk. Parents who are dealing with depression, substance use disorders, or other mental health challenges may be less emotionally available to their children, fostering an environment of emotional neglect (Mondi et al., 2021). Additionally, parental suicide or suicide attempts significantly increase the likelihood of similar behaviors in their children, likely due to both genetic predispositions and environmental modeling (Franklin et al., 2017).

Negative peer experiences like exclusion, rejection, or bullying significantly increase suicide risk. Bullying, in particular, is closely associated with suicidal thoughts and behaviors. Adolescents who are bullied often endure chronic humiliation, fear, and isolation, leading to feelings of hopelessness and despair (Hinduja and Patchin, 2010). Bullying and cyberbullying represent relational stressors, as they directly involve peer interactions and rejection experiences.

Cyberbullying has emerged as a distinct and powerful relational risk factor in the digital age. Unlike traditional bullying, cyberbullying is widespread and unavoidable, as it frequently occurs in spaces where adolescents seek connection, like social media platforms. Victims of cyberbullying report higher levels of depression, anxiety, and suicidal thoughts compared to their peers (Zhou et al., 2023).

Perpetrators of cyberbullying are also at an increased risk of poor mental health outcomes, including suicidal behaviors, suggesting a bidirectional relationship between bullying dynamics and emotional distress. Interventions focusing on digital literacy and promoting respectful online behavior are essential components of suicide prevention efforts (Weersing et al., 2017).

#### 3.1.3 Socio-ecological factors

The accessibility and quality of mental health services are critical systemic factors affecting adolescent suicide risk. Many adolescents, particularly those in underserved or rural areas, face significant barriers to accessing mental health care, including a shortage of mental health professionals, long waiting times, and financial constraints (Cramer and Kapusta, 2017). Moreover, even in areas where services are available, the stigma associated with seeking mental health care often prevents adolescents and families from getting the support they need (Dubé et al., 2014).

Healthcare systems often lack suicide prevention strategies specifically designed for adolescents. Training for healthcare providers to identify and address suicidal behavior in this age group remains inconsistent, resulting in missed opportunities for early intervention (Liu et al., 2024). Integrating mental health services into primary care and schools has shown the potential to improve access and outcomes (Miller-Graff et al., 2016).

Schools play a vital role in shaping adolescent well-being, yet systemic shortcomings frequently undermine their potential as protective environments. Academic pressure and an excessive focus on performance can lead to stress, anxiety, and feelings of inadequacy among adolescents, particularly in competitive educational systems (Lee et al., 2024).

Bullying, including cyberbullying, is a systemic issue in schools that significantly contributes to the risk of suicide. Although many educational institutions have anti-bullying policies, their enforcement and effectiveness vary widely, leaving some adolescents vulnerable to ongoing harassment (Brent et al., 2013). At the same time, bullying and cyberbullying are influenced by systemic determinants such as school policies, digital platform dynamics, and community norms, which sustain or mitigate their prevalence.

Socioeconomic status is a well-documented systemic factor that influences adolescent mental health and suicide risk. Adolescents from low-income families are more likely to face chronic stress, food insecurity, housing instability, and limited access to healthcare and educational resources (King et al., 2018). These stressors intensify feelings of hopelessness and diminish opportunities for positive developmental experiences.

Communities with high unemployment rates or economic hardship often encounter additional systemic challenges, such as underfunded schools, a shortage of recreational facilities, and elevated crime rates, which indirectly increase the risk of adolescent suicide (National Institute of Mental Health, 2023).

### 3.2 Protective factors for adolescent mental health and suicide prevention

Protective factors play a crucial role in mitigating the risk of suicide among adolescents by enhancing resilience, promoting adaptive coping mechanisms, and fostering supportive environments. These factors can be categorized into individual, relational, and systemic domains, offering a multidimensional framework for prevention.

#### 3.2.1 Familial and relational support

Strong parental support and open communication are among the most robust protective factors against adolescent suicide. Adolescents who feel understood and supported by their parents are more likely to seek help during emotional crises (Ho et al., 2022). Parenting practices that include active listening, encouragement, and shared decision-making contribute to increased self-esteem and emotional regulation (Cox and Hetrick, 2017). Family-based interventions that improve communication and mental health literacy have demonstrated effectiveness in reducing suicide risk (Victor et al., 2023).

#### 3.2.2 Peer support and social connectedness

Supportive peer relationships serve as protective buffers by offering emotional validation, reducing loneliness, and encouraging help-seeking behavior. Adolescents with strong, positive social networks exhibit better stress management and lower levels of psychological distress (Solstad et al., 2021). Peer-led programs and mentorship initiatives can reinforce connectedness and promote resilience.

#### 3.2.3 School and community resources

Schools that implement social–emotional learning (SEL) programs, anti-bullying policies, and mental health services are better positioned to support adolescent well-being and prevent suicide (Bas, 2021; Heppen et al., 2018). School counselors and mental health professionals play a pivotal role in early detection and intervention. At the community level, access to youth centers, extracurricular activities, and culturally sensitive outreach programs enhances protective support.

#### 3.2.4 Individual traits and coping skills

On an individual level, traits such as emotional intelligence, self-efficacy, and optimism contribute to a reduced risk of suicidal behavior. Psychoeducational and skill-building interventions that teach adolescents coping strategies, emotional regulation, and problem-solving can bolster their resilience. Mindfulness-based approaches and cognitive restructuring techniques have shown promise in promoting mental wellness and reducing impulsive behavior.

Together, these protective factors offer a foundation for comprehensive suicide prevention strategies that extend beyond clinical settings to include families, schools, and communities (Weersing et al., 2017; Sultan et al., 2025).

### 3.3 Prevention strategies

Cognitive-behavioral therapy (CBT) is one of the most evidence-based methods for addressing suicidal thoughts and behaviors in adolescents. It emphasizes the identification and modification of negative thought patterns, enhances problem-solving skills, and teaches adaptive coping strategies. Adolescents undergoing CBT learn to reframe cognitive distortions, such as feelings of hopelessness or worthlessness, which are closely linked to suicidal ideation (Keles and Idsoe, 2018).

cognitive behavioral therapy for suicide prevention (CBT-SP) is a specialized approach that incorporates safety planning and emotional regulation strategies to effectively address suicidal behaviors. Research indicates that CBT-SP significantly decreases both suicide attempts and ideation among high-risk adolescents (Brown et al., 2005).

Dialectical Behavior Therapy (DBT), which was originally developed for individuals with borderline personality disorder, has been adapted for adolescents and proves to be especially effective for those at high risk of suicide. DBT integrates individual therapy, skills training, and family involvement to address emotional dysregulation, a common factor in suicidal behaviors (Mehlum et al., 2014).

Key components of DBT for adolescents involve teaching mindfulness, distress tolerance, emotion regulation, and interpersonal effectiveness. The therapy also incorporates crisis intervention strategies, such as developing a safety plan and enhancing caregiver communication (McCauley et al., 2018). Research shows that DBT decreases suicidal ideation, self-harm behaviors, and psychiatric hospitalizations in adolescents (Santamarina-Perez et al., 2020).

Pharmacological treatment is often necessary for adolescents with underlying psychiatric conditions contributing to suicide risk, such as depression, anxiety, or bipolar disorder. Selective serotonin reuptake inhibitors (SSRIs) are commonly prescribed for depression and have shown efficacy in reducing symptoms associated with suicidal ideation (Young et al., 2010).

However, careful monitoring is essential because of the potential for increased agitation or suicidal thoughts during the initial weeks of SSRI treatment. Regular follow-up appointments and open communication with the adolescent and their family are crucial for ensuring the safe and effective use of medications (Emslie et al., 2010).

For treating depression that resists other treatments, interventions such as esketamine nasal spray have been studied, showing potential for rapidly decreasing suicidal thoughts. Current research is focused on confirming its safety and effectiveness in adolescents (Witt et al., 2021).

Psychoeducation serves as a cornerstone of individual-level interventions, equipping adolescents with crucial knowledge about mental health, suicide risk, and coping strategies. They learn to recognize early signs of distress, understand their triggers, and implement proactive coping mechanisms (Kemper et al., 2021).

Skill-building programs teach adolescents practical techniques for managing stress, regulating emotions, and improving problem-solving skills. For example, mindfulness-based interventions foster self-awareness and reduce impulsivity, both of which are vital in preventing suicide attempts (Witt et al., 2021).

## 4 Discussion

**Figure 3:**
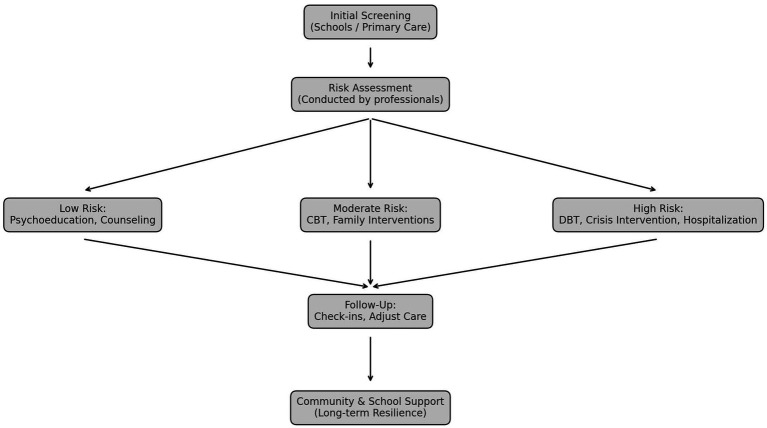
Stepped-care framework for adolescent suicide prevention. Initial screening occurs in schools or primary care, followed by professional risk assessment. According to risk level, interventions include psychoeducation and counseling for low risk, CBT and family interventions for moderate risk, and DBT, crisis intervention, or hospitalization for high risk. Ongoing follow-up, community, and school-based support promote long-term resilience.

This narrative review synthesizes a wide array of evidence on adolescent suicide, providing a multidimensional perspective that encompasses individual, relational, and systemic domains. The findings reaffirm the multifactorial nature of suicide risk, shaped by the interaction of mental health disorders, adverse life experiences, family dynamics, peer influences, and broader socio-environmental factors (Carballo et al., 2020; Hink et al., 2022). The effectiveness of various prevention strategies—ranging from evidence-based psychotherapies to school and community-based initiatives—is promising; however, significant challenges remain in translating this knowledge into equitable and scalable interventions (Caro-Cañizares et al., 2024). In particular, adolescents living with parents affected by mental illness represent a vulnerable and often overlooked group. These young people may experience role reversal and assume caregiving responsibilities, which can compromise their development and well-being (Reupert et al., 2013). For this population, preventive actions should prioritize early identification and family-based interventions, including programs that promote parental mental health literacy, strengthen communication within the family, and provide external psychosocial and caregiving support (Müller et al., 2024). Such approaches may reduce the caregiving burden on adolescents and foster resilience in the face of adversity. Building on the evidence reviewed, Figure 3 presents a stepped-care framework for adolescent suicide prevention, outlining how screening, risk assessment, and tailored interventions can be integrated with long-term community and school support.

### 4.1 Critical gaps and unmet needs

Despite decades of research, substantial gaps remain in both our understanding and ability to prevent adolescent suicide. One pressing issue is the lack of longitudinal studies that can elucidate the developmental trajectories and causal mechanisms underlying suicidal behavior. Most existing evidence is cross-sectional, which limits the capacity to distinguish between correlates and predictors of suicide risk.

Furthermore, there is limited research on the effectiveness of interventions in real-world settings, especially for high-risk or underserved populations. Adolescents facing structural inequalities—such as poverty, discrimination, chronic illness, or community violence—are largely underrepresented in prevention trials. This raises questions about the generalizability of current evidence and underscores the urgent need for more inclusive research designs.

Another critical unmet need involves the integration of digital health tools. While smartphone apps, telepsychiatry, and AI-driven interventions present new opportunities for early detection and support, few have undergone rigorous evaluation, and concerns regarding privacy, equity, and engagement remain unresolved. Additionally, the field lacks guidelines for the ethical and effective use of technology-based interventions in adolescent populations.

Finally, there is a lack of research focusing on protective factors such as emotional resilience, connectedness, and supportive family and school environments. While these factors are often mentioned, they are rarely investigated as primary outcomes in clinical and community-based studies. Shifting the focus from risk mitigation to promoting resilience could improve the effectiveness and acceptability of suicide prevention efforts.

### 4.2 Strengths and contributions of this review

A key strength of this review lies in its integrative framework, which examines adolescent suicide through a multilevel lens that encompasses clinical, familial, educational, and policy-oriented perspectives. This holistic approach captures the complexity of suicide risk and aligns with contemporary calls for transdisciplinary collaboration in prevention efforts.

Unlike many earlier reviews that concentrate solely on clinical interventions, this work also emphasizes relational and systemic factors, such as parenting practices, peer dynamics, school climate, and socioeconomic determinants. Furthermore, the review includes emerging evidence on digital technologies, an area of growing significance given adolescents’ engagement with online platforms.

By merging empirical findings with conceptual insights, the review provides a valuable resource for clinicians, educators, and policymakers, while establishing a foundation for the development of comprehensive, context-sensitive prevention programs.

### 4.3 Limitations of the current literature

The current body of research is characterized by several limitations. Many studies exhibit methodological heterogeneity, including variations in definitions of suicidal ideation and behavior, inconsistent outcome measures, and differing time frames. This heterogeneity complicates comparisons across studies and hinders the synthesis of strong conclusions.

Moreover, there is a significant scarcity of randomized controlled trials (RCTs) specifically focused on suicide prevention in adolescents. The few available RCTs often lack adequate follow-up periods, which limits understanding of the long-term efficacy of interventions. Additionally, there is insufficient data on cost-effectiveness, posing challenges for policy implementation in resource-limited settings.

Ethical and logistical concerns also play a role in the underrepresentation of adolescents in research, especially those considered to be at the highest risk. As a result, the perspectives of the most vulnerable youths are frequently absent from the evidence base.

## 5 Limitations of this review

As a narrative review, this article has several inherent limitations. The lack of a systematic search protocol and formal quality appraisal may introduce selection bias and reduce replicability. Although efforts were made to include high-quality and recent studies, the breadth of the literature could have led to the omission of some relevant findings. Furthermore, the narrative format, while flexible and integrative, does not facilitate quantitative synthesis or meta-analytical interpretation.

## 6 Conclusion and outlook

Adolescent suicide is a complex and multifaceted issue that requires a comprehensive approach integrating individual, relational, and systemic interventions. This narrative review emphasizes the interplay of personal vulnerabilities, family dynamics, peer relationships, societal influences, and structural barriers in shaping suicide risk among adolescents. While significant progress has been made in understanding and addressing these factors, critical gaps still require focused attention.

Individual-level interventions, like CBT, DBT, and safety planning, have proven effective in reducing suicidal behaviors. However, their accessibility is often limited by systemic issues, such as a shortage of trained professionals and unequal distribution of mental health resources. Relational interventions stress the vital role of families, peers, and educators in supporting adolescents. Yet, the success of these efforts largely depends on tackling broader systemic challenges, including bullying, stigma, and socioeconomic disparities.

Systemic interventions, such as policy reforms, school-based mental health programs, and community-driven initiatives, offer a pathway to sustainable change. Nevertheless, to ensure their reach and impact, these efforts require consistent funding, cross-sector collaboration, and cultural sensitivity. Unlike previous reviews on adolescent suicide, our work integrates individual, familial, and systemic perspectives, offering a comprehensive framework applicable to diverse healthcare and educational settings. Additionally, it updates the literature by including recent studies on the role of digital technologies in prevention.

## 7 Future directions

Moving forward, suicide prevention in adolescents requires coordinated efforts across clinical, family, educational, and policy areas. Key priorities include:

1. Technology and digital innovations: Mobile apps, telehealth services, and artificial intelligence can increase access and enable early detection through real-time monitoring and psychoeducation. To maximize impact, future research should focus on usability, cultural adaptation, and equitable access.
2. Community and school-based models: Community-led initiatives and school mental health programs are vital for reaching adolescents who may not seek formal care. Collaborations with teachers, peer mentors, and youth advocates can foster supportive environments and lessen stigma.
3. Policy and advocacy: Sustainable funding and policy reforms are crucial for strengthening suicide prevention systems. Priorities include enforcing anti-bullying policies, improving access to care, and supporting families through workplace and social policies.
4. High-risk and underserved groups: Customized interventions are essential for adolescents exposed to disproportionate risks, including those living in poverty, facing discrimination, or managing chronic illnesses. Addressing these disparities is vital to promote fairness in outcomes.
5. Strengthening families and building resilience: Family-centered programs that improve communication and parenting skills, along with interventions that promote coping, emotional regulation, and connectedness, can lower vulnerability and help adolescents face challenges.

### 7.1 A call to action

Preventing adolescent suicide is both a clinical challenge and a societal responsibility. Without strong efforts, disparities in access and outcomes will continue. Collaboration among healthcare providers, educators, policymakers, and communities is urgently needed to remove barriers, build resilience, and encourage help-seeking. By focusing on adolescent well-being and integrating suicide prevention into schools, families, and communities, we can create environments where young people feel supported, valued, and prepared to succeed.
